# Tris(1,10-phenanthroline-κ^2^ *N*,*N*′)ruthenium(II) bis­(perchlorate)

**DOI:** 10.1107/S1600536812048428

**Published:** 2012-11-30

**Authors:** Mariana Kozlowska, Pawel Rodziewicz, Diana Malgorzata Brus, Justyna Czyrko, Krzysztof Brzezinski

**Affiliations:** a Institute of Chemistry, University of Bialystok, Hurtowa 1, 15-399 Bialystok, Poland

## Abstract

The asymmetric unit of the title compound, [Ru(C_12_H_8_N_2_)_3_](ClO_4_)_2_, contains one octahedrally coordinated Ru^II^ cation of the ruthenium-phenanthroline complex and three differently occupied perchlorate anions: two, denoted *A* and *B*, are located on the twofold axis while another, denoted *C*, is positioned in the proximity of the twofold screw axis. Perchlorate anions *B* and *C* are severely disordered. The occupancies of the two major conformers of anion *B* refined to 0.302 (6) and 0.198 (6). Perchlorate ion *C* was modeled in two alternate conformations which refined to occupancies of 0.552 (10) and 0.448 (10).

## Related literature

For the preparation of phenanthroline complexes with transition metals, see: Burstall & Nyholm (1952 ▶). For the structures of salts of complexes of ruthenium with phenanthroline, see: Breu & Stoll (1996 ▶); Maloney & MacDonnell (1997 ▶); Otsuka *et al.* (2001 ▶); Wu *et al.* (2001 ▶); Ghazzali *et al.* (2008 ▶). For background to the properties and applications of phenanthroline complexes, see: Juris *et al.* (1988 ▶); D’Angelantonio *et al.* (1991 ▶); Balzani *et al.* (1996 ▶); Mills & Williams (1997 ▶); Yang *et al.* (1997 ▶); Miyasaka *et al.* (2001 ▶); Plonska *et al.* (2002 ▶); Winkler *et al.* (2006 ▶).
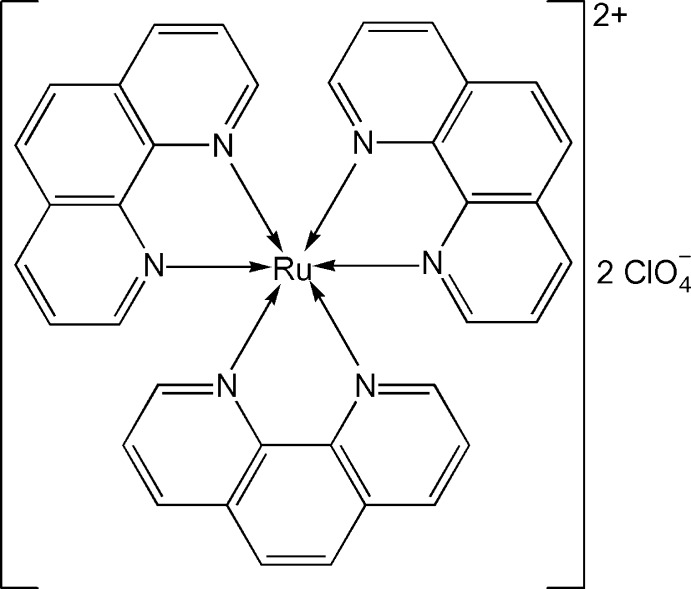


## Experimental

### 

#### Crystal data

[Ru(C_12_H_8_N_2_)_3_](ClO_4_)_2_
*M* *_r_* = 840.57
Monoclinic, 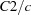

*a* = 35.408 (7) Å
*b* = 16.106 (3) Å
*c* = 12.056 (2) Å
β = 102.22 (3)°
*V* = 6720 (2) Å^3^
*Z* = 8
Mo *K*α radiation
μ = 0.69 mm^−1^
*T* = 100 K
0.22 × 0.19 × 0.10 mm

#### Data collection

Agilent SuperNova (Dual, Cu at zero, Atlas) diffractometer
Absorption correction: multi-scan (*CrysAlis PRO*; Agilent, 2011 ▶) *T*_min_ = 0.859, *T*_max_ = 1.000
28067 measured reflections
6867 independent reflections
5365 reflections with *I* > 2σ(*I*)
*R*_int_ = 0.039

#### Refinement

*R*[*F* ^2^ > 2σ(*F* ^2^)] = 0.071
*wR*(*F* ^2^) = 0.218
*S* = 1.04
6867 reflections
545 parameters
181 restraints
H-atom parameters constrained
Δρ_max_ = 2.55 e Å^−3^
Δρ_min_ = −1.22 e Å^−3^

### 

Data collection: *CrysAlis PRO* (Agilent, 2011 ▶); cell refinement: *CrysAlis PRO*; data reduction: *CrysAlis PRO*; program(s) used to solve structure: *SHELXD* (Sheldrick, 2008 ▶); program(s) used to refine structure: *SHELXL97* (Sheldrick, 2008 ▶); molecular graphics: *ORTEP-3* (Farrugia, 2012) ▶; software used to prepare material for publication: *SHELXL97*.

## Supplementary Material

Crystal structure: contains datablock(s) global, I. DOI: 10.1107/S1600536812048428/bx2430sup1.cif

Structure factors: contains datablock(s) I. DOI: 10.1107/S1600536812048428/bx2430Isup2.hkl

Additional supplementary materials:  crystallographic information; 3D view; checkCIF report

## Figures and Tables

**Fig. 1.**
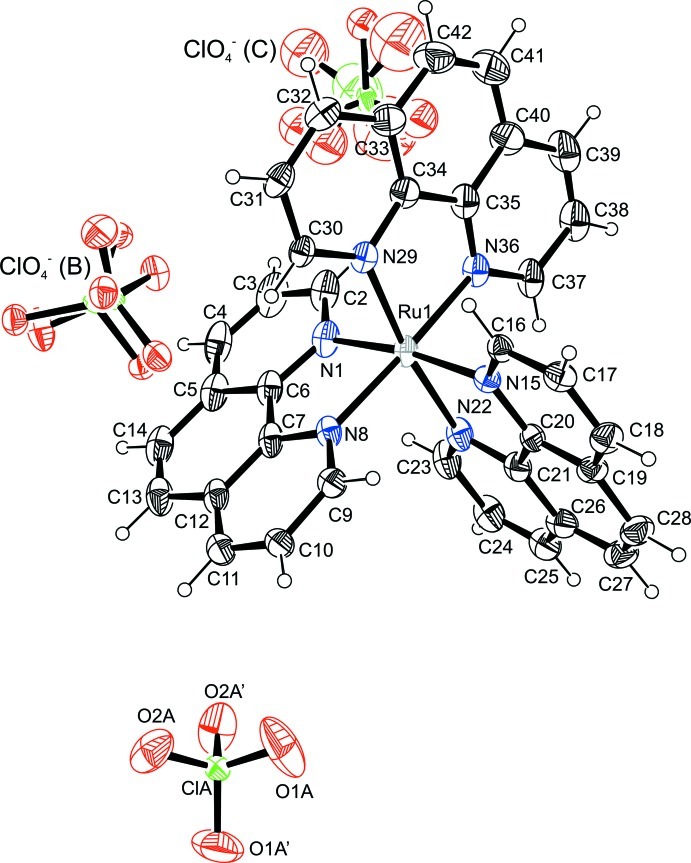
The molecular structure of the title compound. Displacement ellipsoids are drawn at the 30% probability level. For clarity, labels for atoms in the disordered perchlorate anions B and C are omitted.

## supplementary crystallographic information

### Comment

1,10-Phenanthroline (phen), forms complexes with most of transition metals. The polyimine complexes of divalent transition metal cations, such as [Ru^II^(phen)_3_](ClO_4_)_2_ or [Ru^II^(bpy)_3_](ClO_4_)_2_ (bpy-2,2'-bipyridine), are well known potent photosensitizers (Juris *et al.*, 1988). These compounds reveal also other interesting properties due to their redox (Plonska *et al.*, 2002; Winkler *et al.*, 2006) and magnetic properties (Miyasaka *et al.*, 2001), excited-state reactivity (D'Angelantonio *et al.*, 1991), and emission and lifetime characteristics (Juris *et al.*, 1988; Balzani *et al.*, 1996). A high photostability, long excited-state lifetimes and high quantum yields of luminescence, enabled to use them as oxygen optical sensors (Mills *et al.*, 1997). A binding of these complexes to calf thymus DNA has been also investigated (Yang *et al.*, 1997).

The asymmetric unit contains one divalent cation of the ruthenium-phenanthroline complex and three differently occupied perchlorate anions (Fig. 1). The half-ion of perchlorate A is located on the twofold axis and the complete anion is generated by the symmetry operation. Perchlorate anions, B and C are disordered and each one of them is modeled in two alternative conformations. The occupancy of two major conformers is refined to 0.302 (6) and 0.198 (6) or 0.552 (10) and 0.448 (10) for anion B or C, respectively. Conformers of perchlorate ion B are located on the twofold axis.

### Experimental

The transition metal complex salt, [Ru^II^(phen)_3_](ClO_4_)_2_ was prepared according to the procedure described by Burstall *et al.*, 1952 and was recrystallized from methanol.

### Refinement

The solvent/anion region is highly disordered and the final difference minimum and maximum (-1.15 and 2.62 e Å^-3^) indicate an its imperfect modeling. The highest difference peak corresponds to solvent accessible void in the crystal lattice. The disordered perchlorate anion B and C are modeled in two alternative conformations with geometric restraints (*DFIX* and *SADI* instructions). Additionally, displacement parameter restraints (*DELU* and *ISOR* instructions) are applied for anion B. Due to a serious disorder of perchlorate anion C, its oxygen atoms are refined isotropically. All H atoms were located in electron density difference maps. C-bonded hydrogen atoms were constrained to idealized positions with C—H distances fixed at 0.95 Å and 1.2*U*_eq_(C).

### Crystal data

**Table:**

[Ru(C_12_H_8_N_2_)_3_](ClO_4_)_2_	*F*(000) = 3392
*M**_r_* = 840.57	*D*_x_ = 1.662 Mg m^−^^3^
Monoclinic, *C*2/*c*	Mo *K*α radiation, λ = 0.71073 Å
Hall symbol: -C 2yc	Cell parameters from 28661 reflections
*a* = 35.408 (7) Å	θ = 2.6–26.3°
*b* = 16.106 (3) Å	µ = 0.69 mm^−^^1^
*c* = 12.056 (2) Å	*T* = 100 K
β = 102.22 (3)°	Plate, red
*V* = 6720 (2) Å^3^	0.22 × 0.19 × 0.10 mm
*Z* = 8	

### Data collection

**Table:**

Agilent SuperNova (Dual, Cu at zero, Atlas) diffractometer	6867 independent reflections
Radiation source: SuperNova (Mo) X-ray Source	5365 reflections with *I* > 2σ(*I*)
Mirror monochromator	*R*_int_ = 0.039
Detector resolution: 10.4052 pixels mm^-1^	θ_max_ = 26.4°, θ_min_ = 2.8°
ω scans	*h* = −44→44
Absorption correction: multi-scan (*CrysAlis PRO*; Agilent, 2011)	*k* = −20→20
*T*_min_ = 0.859, *T*_max_ = 1.000	*l* = −14→15
28067 measured reflections	

### Refinement

**Table:**

Refinement on *F*^2^	Primary atom site location: structure-invariant direct methods
Least-squares matrix: full	Secondary atom site location: difference Fourier map
*R*[*F*^2^ > 2σ(*F*^2^)] = 0.071	Hydrogen site location: inferred from neighbouring sites
*wR*(*F*^2^) = 0.218	H-atom parameters constrained
*S* = 1.04	*w* = 1/[σ^2^(*F*_o_^2^) + (0.1194*P*)^2^ + 53.7304*P*] where *P* = (*F*_o_^2^ + 2*F*_c_^2^)/3
6867 reflections	(Δ/σ)_max_ = 0.001
545 parameters	Δρ_max_ = 2.55 e Å^−^^3^
181 restraints	Δρ_min_ = −1.22 e Å^−^^3^

### Special details

**Table:**

Geometry. All e.s.d.'s (except the e.s.d. in the dihedral angle between two l.s. planes) are estimated using the full covariance matrix. The cell e.s.d.'s are taken into account individually in the estimation of e.s.d.'s in distances, angles and torsion angles; correlations between e.s.d.'s in cell parameters are only used when they are defined by crystal symmetry. An approximate (isotropic) treatment of cell e.s.d.'s is used for estimating e.s.d.'s involving l.s. planes.
Refinement. Refinement of *F*^2^ against ALL reflections. The weighted *R*-factor *wR* and goodness of fit *S* are based on *F*^2^, conventional *R*-factors *R* are based on *F*, with *F* set to zero for negative *F*^2^. The threshold expression of *F*^2^ > σ(*F*^2^) is used only for calculating *R*-factors(gt) *etc*. and is not relevant to the choice of reflections for refinement. *R*-factors based on *F*^2^ are statistically about twice as large as those based on *F*, and *R*- factors based on ALL data will be even larger.

### Fractional atomic coordinates and isotropic or equivalent isotropic displacement parameters (Å^2^)

**Table:**

	*x*	*y*	*z*	*U*_iso_*/*U*_eq_	Occ. (<1)
ClA	0.5000	0.08518 (12)	1.2500	0.0409 (4)	
O1A	0.4920 (2)	0.0360 (6)	1.1532 (7)	0.137 (4)	
O2A	0.53245 (17)	0.1346 (4)	1.2483 (7)	0.098 (2)	
ClBA	0.49125 (14)	0.4508 (3)	0.7332 (5)	0.0377 (19)	0.302 (6)
O1BA	0.45000 (16)	0.4625 (5)	0.7094 (9)	0.048 (3)	0.302 (6)
O2BA	0.5065 (3)	0.4809 (5)	0.6390 (9)	0.062 (5)	0.302 (6)
O3BA	0.5000	0.3639 (3)	0.7500	0.060 (3)	0.605 (13)
O4BA	0.5085 (3)	0.4961 (5)	0.8342 (8)	0.064 (5)	0.302 (6)
ClBB	0.5000	0.4583 (5)	0.7500	0.032 (2)	0.395 (13)
O1BB	0.5200 (4)	0.5161 (10)	0.8333 (10)	0.046 (4)	0.198 (6)
O2BB	0.4835 (3)	0.3927 (7)	0.8057 (11)	0.035 (4)	0.198 (6)
O3BB	0.5266 (3)	0.4238 (8)	0.6871 (10)	0.044 (4)	0.198 (6)
O4BB	0.4697 (3)	0.5022 (12)	0.6740 (12)	0.047 (4)	0.198 (6)
ClCA	0.26318 (8)	0.5086 (2)	0.4468 (2)	0.0435 (11)	0.552 (10)
O1CA	0.2538 (2)	0.5648 (4)	0.3520 (5)	0.064 (3)*	0.552 (10)
O2CA	0.23054 (19)	0.5010 (6)	0.4998 (7)	0.277 (19)*	0.552 (10)
O3CA	0.2726 (3)	0.4284 (3)	0.4073 (7)	0.091 (4)*	0.552 (10)
O4CA	0.29584 (19)	0.5403 (5)	0.5278 (6)	0.092 (4)*	0.552 (10)
ClCB	0.2615 (2)	0.5258 (6)	0.4462 (7)	0.166 (5)	0.448 (10)
O1CB	0.2576 (6)	0.6144 (5)	0.4546 (17)	0.149 (9)*	0.448 (10)
O2CB	0.2292 (2)	0.4858 (6)	0.4795 (8)	0.049 (3)*	0.448 (10)
O3CB	0.2619 (4)	0.5039 (12)	0.3306 (8)	0.212 (14)*	0.448 (10)
O4CB	0.2969 (2)	0.4984 (13)	0.5192 (13)	0.117 (6)*	0.448 (10)
Ru1	0.379433 (12)	0.21680 (3)	0.62688 (4)	0.03927 (19)	
N8	0.42571 (13)	0.2140 (3)	0.7640 (4)	0.0407 (11)	
N36	0.33253 (14)	0.2267 (3)	0.4936 (5)	0.0455 (12)	
N29	0.40257 (13)	0.2905 (3)	0.5175 (4)	0.0404 (11)	
N22	0.35194 (13)	0.1363 (4)	0.7156 (4)	0.0475 (12)	
C26	0.33993 (17)	−0.0117 (5)	0.7393 (6)	0.0522 (16)	
N1	0.36793 (14)	0.3206 (4)	0.7139 (5)	0.0515 (13)	
C31	0.44900 (18)	0.3752 (4)	0.4513 (6)	0.0509 (15)	
H31	0.4740	0.3998	0.4661	0.061*	
C25	0.31696 (19)	0.0092 (5)	0.8174 (6)	0.0602 (19)	
H25	0.3044	−0.0332	0.8510	0.072*	
C17	0.42222 (17)	0.0127 (4)	0.4478 (5)	0.0454 (13)	
H17	0.4373	0.0055	0.3921	0.054*	
C7	0.42374 (17)	0.2736 (4)	0.8443 (5)	0.0468 (15)	
C16	0.41419 (14)	0.0922 (4)	0.4812 (4)	0.0360 (11)	
H16	0.4239	0.1385	0.4470	0.043*	
N15	0.39328 (12)	0.1058 (3)	0.5599 (4)	0.0367 (10)	
C35	0.33975 (18)	0.2702 (4)	0.4047 (6)	0.0510 (16)	
C30	0.43730 (16)	0.3264 (3)	0.5347 (5)	0.0403 (12)	
H30	0.4547	0.3187	0.6056	0.048*	
C27	0.3471 (2)	−0.0933 (5)	0.7063 (6)	0.0586 (18)	
H27	0.3358	−0.1384	0.7386	0.070*	
C11	0.4786 (2)	0.2177 (4)	0.9732 (5)	0.0539 (17)	
H11	0.4967	0.2179	1.0437	0.065*	
C37	0.29761 (16)	0.1926 (4)	0.4833 (7)	0.0560 (17)	
H37	0.2924	0.1608	0.5448	0.067*	
C39	0.2751 (2)	0.2466 (6)	0.2965 (8)	0.077 (2)	
H39	0.2554	0.2533	0.2301	0.093*	
C12	0.4499 (2)	0.2781 (4)	0.9494 (6)	0.0521 (17)	
C40	0.3120 (2)	0.2835 (5)	0.3033 (7)	0.066 (2)	
C21	0.35678 (15)	0.0539 (4)	0.6894 (5)	0.0441 (13)	
C41	0.3230 (3)	0.3303 (6)	0.2160 (8)	0.084 (3)	
H41	0.3045	0.3397	0.1476	0.101*	
C18	0.40855 (18)	−0.0547 (4)	0.4948 (6)	0.0501 (15)	
H18	0.4141	−0.1092	0.4724	0.060*	
C14	0.4168 (2)	0.4011 (5)	0.9966 (7)	0.067 (2)	
H14	0.4150	0.4452	1.0476	0.080*	
C2	0.33910 (19)	0.3754 (5)	0.6835 (8)	0.063 (2)	
H2	0.3223	0.3709	0.6111	0.076*	
C4	0.3582 (2)	0.4498 (5)	0.8596 (8)	0.071 (2)	
H4	0.3542	0.4937	0.9083	0.086*	
C9	0.45398 (16)	0.1585 (4)	0.7900 (5)	0.0444 (13)	
H9	0.4562	0.1173	0.7354	0.053*	
C13	0.4454 (2)	0.3449 (5)	1.0251 (6)	0.063 (2)	
H13	0.4628	0.3493	1.0964	0.075*	
C6	0.39295 (17)	0.3319 (4)	0.8162 (6)	0.0493 (15)	
C19	0.38602 (16)	−0.0429 (4)	0.5768 (5)	0.0451 (14)	
C20	0.37928 (14)	0.0384 (4)	0.6065 (5)	0.0380 (12)	
C38	0.26831 (19)	0.2015 (5)	0.3861 (8)	0.067 (2)	
H38	0.2438	0.1764	0.3825	0.081*	
C24	0.31276 (18)	0.0892 (5)	0.8445 (6)	0.0612 (19)	
H24	0.2978	0.1032	0.8985	0.073*	
C42	0.3588 (3)	0.3619 (6)	0.2264 (8)	0.079 (2)	
H42	0.3651	0.3920	0.1649	0.095*	
C33	0.3874 (2)	0.3510 (4)	0.3275 (6)	0.0586 (17)	
C23	0.33056 (17)	0.1525 (5)	0.7930 (6)	0.0581 (18)	
H23	0.3273	0.2085	0.8137	0.070*	
C28	0.3692 (2)	−0.1095 (4)	0.6305 (6)	0.0595 (18)	
H28	0.3738	−0.1655	0.6123	0.071*	
C5	0.3892 (2)	0.3961 (5)	0.8918 (6)	0.0566 (17)	
C10	0.48045 (17)	0.1579 (4)	0.8934 (5)	0.0486 (15)	
H10	0.4998	0.1161	0.9087	0.058*	
C32	0.4242 (2)	0.3874 (4)	0.3480 (7)	0.0584 (17)	
H32	0.4319	0.4201	0.2911	0.070*	
C34	0.37750 (17)	0.3047 (4)	0.4163 (6)	0.0481 (14)	
C3	0.3336 (2)	0.4388 (5)	0.7571 (8)	0.074 (2)	
H3	0.3122	0.4752	0.7354	0.088*	

### Atomic displacement parameters (Å^2^)

**Table:**

	*U*^11^	*U*^22^	*U*^33^	*U*^12^	*U*^13^	*U*^23^
ClA	0.0410 (10)	0.0385 (10)	0.0390 (10)	0.000	−0.0006 (8)	0.000
O1A	0.106 (5)	0.182 (9)	0.104 (5)	0.037 (6)	−0.021 (4)	−0.086 (6)
O2A	0.059 (3)	0.079 (4)	0.161 (7)	−0.008 (3)	0.035 (4)	0.029 (4)
ClBA	0.045 (3)	0.035 (3)	0.029 (3)	−0.007 (2)	−0.002 (3)	0.001 (2)
O1BA	0.040 (5)	0.052 (7)	0.055 (7)	0.002 (5)	0.012 (5)	−0.014 (6)
O2BA	0.053 (7)	0.079 (8)	0.060 (7)	−0.014 (7)	0.027 (6)	0.010 (6)
O3BA	0.068 (6)	0.043 (4)	0.055 (6)	0.000	−0.017 (5)	0.000
O4BA	0.053 (8)	0.074 (8)	0.066 (7)	−0.010 (7)	0.012 (6)	−0.033 (7)
ClBB	0.050 (4)	0.026 (3)	0.016 (3)	0.000	−0.004 (3)	0.000
O1BB	0.050 (6)	0.043 (6)	0.043 (5)	−0.009 (4)	0.008 (4)	−0.007 (4)
O2BB	0.033 (6)	0.035 (5)	0.036 (6)	0.002 (4)	0.008 (4)	0.002 (4)
O3BB	0.043 (6)	0.046 (6)	0.045 (6)	−0.007 (4)	0.015 (4)	−0.007 (4)
O4BB	0.051 (6)	0.045 (6)	0.043 (5)	0.007 (4)	0.005 (4)	0.006 (4)
ClCA	0.0446 (18)	0.0573 (19)	0.0324 (15)	−0.0289 (13)	0.0166 (12)	−0.0096 (11)
ClCB	0.112 (7)	0.231 (10)	0.155 (8)	−0.029 (7)	0.029 (6)	−0.011 (7)
Ru1	0.0236 (3)	0.0438 (3)	0.0511 (3)	−0.00316 (17)	0.00957 (19)	−0.0083 (2)
N8	0.029 (2)	0.052 (3)	0.044 (3)	−0.011 (2)	0.0141 (19)	−0.007 (2)
N36	0.027 (2)	0.046 (3)	0.062 (3)	0.0037 (19)	0.006 (2)	−0.009 (2)
N29	0.031 (2)	0.036 (2)	0.055 (3)	0.0039 (18)	0.011 (2)	−0.005 (2)
N22	0.027 (2)	0.062 (3)	0.054 (3)	−0.011 (2)	0.011 (2)	−0.008 (3)
C26	0.033 (3)	0.069 (4)	0.052 (4)	−0.009 (3)	0.005 (3)	0.012 (3)
N1	0.033 (2)	0.054 (3)	0.072 (4)	−0.006 (2)	0.022 (2)	−0.015 (3)
C31	0.044 (3)	0.036 (3)	0.076 (4)	0.002 (2)	0.020 (3)	−0.001 (3)
C25	0.041 (3)	0.083 (5)	0.056 (4)	−0.019 (3)	0.010 (3)	0.008 (4)
C17	0.039 (3)	0.049 (3)	0.047 (3)	0.009 (3)	0.008 (2)	0.002 (3)
C7	0.036 (3)	0.057 (4)	0.053 (3)	−0.020 (3)	0.021 (3)	−0.013 (3)
C16	0.029 (2)	0.039 (3)	0.038 (3)	0.001 (2)	0.003 (2)	−0.001 (2)
N15	0.026 (2)	0.041 (2)	0.042 (2)	0.0037 (18)	0.0020 (18)	0.001 (2)
C35	0.033 (3)	0.045 (3)	0.070 (4)	0.010 (2)	0.001 (3)	−0.008 (3)
C30	0.035 (3)	0.033 (3)	0.054 (3)	0.001 (2)	0.011 (2)	−0.006 (2)
C27	0.048 (4)	0.059 (4)	0.067 (4)	−0.008 (3)	0.008 (3)	0.021 (3)
C11	0.053 (4)	0.074 (5)	0.037 (3)	−0.026 (3)	0.014 (3)	0.002 (3)
C37	0.025 (3)	0.053 (4)	0.085 (5)	0.003 (3)	0.002 (3)	−0.018 (3)
C39	0.041 (4)	0.076 (5)	0.098 (6)	0.019 (4)	−0.021 (4)	−0.015 (4)
C12	0.053 (4)	0.063 (4)	0.045 (3)	−0.030 (3)	0.023 (3)	−0.009 (3)
C40	0.044 (4)	0.062 (5)	0.080 (5)	0.011 (3)	−0.010 (4)	0.012 (4)
C21	0.028 (3)	0.054 (4)	0.049 (3)	−0.006 (2)	0.004 (2)	0.001 (3)
C41	0.071 (5)	0.087 (6)	0.079 (6)	0.010 (5)	−0.022 (4)	0.013 (5)
C18	0.043 (3)	0.043 (3)	0.063 (4)	0.011 (3)	0.006 (3)	0.005 (3)
C14	0.080 (5)	0.068 (5)	0.065 (5)	−0.034 (4)	0.044 (4)	−0.024 (4)
C2	0.039 (3)	0.058 (4)	0.098 (6)	−0.005 (3)	0.026 (3)	−0.023 (4)
C4	0.063 (5)	0.065 (5)	0.097 (6)	−0.023 (4)	0.043 (4)	−0.035 (4)
C9	0.034 (3)	0.055 (4)	0.046 (3)	−0.008 (3)	0.012 (2)	0.003 (3)
C13	0.067 (4)	0.076 (5)	0.052 (4)	−0.028 (4)	0.025 (3)	−0.021 (4)
C6	0.041 (3)	0.056 (4)	0.059 (4)	−0.016 (3)	0.027 (3)	−0.016 (3)
C19	0.036 (3)	0.042 (3)	0.053 (3)	0.006 (2)	0.001 (2)	0.010 (3)
C20	0.026 (2)	0.043 (3)	0.042 (3)	−0.004 (2)	0.000 (2)	0.000 (2)
C38	0.031 (3)	0.062 (4)	0.101 (6)	0.011 (3)	−0.005 (3)	−0.018 (4)
C24	0.035 (3)	0.094 (6)	0.058 (4)	−0.019 (3)	0.019 (3)	−0.007 (4)
C42	0.081 (6)	0.078 (6)	0.071 (5)	0.015 (5)	0.002 (4)	0.022 (4)
C33	0.057 (4)	0.052 (4)	0.065 (4)	0.012 (3)	0.007 (3)	0.006 (3)
C23	0.032 (3)	0.079 (5)	0.066 (4)	−0.014 (3)	0.018 (3)	−0.020 (4)
C28	0.053 (4)	0.046 (4)	0.074 (5)	0.004 (3)	0.000 (3)	0.014 (3)
C5	0.051 (4)	0.060 (4)	0.069 (4)	−0.017 (3)	0.038 (3)	−0.018 (3)
C10	0.041 (3)	0.065 (4)	0.040 (3)	−0.016 (3)	0.007 (2)	0.008 (3)
C32	0.060 (4)	0.047 (4)	0.072 (5)	0.002 (3)	0.022 (4)	0.008 (3)
C34	0.037 (3)	0.041 (3)	0.062 (4)	0.007 (2)	0.004 (3)	0.001 (3)
C3	0.049 (4)	0.059 (4)	0.123 (7)	0.001 (3)	0.040 (4)	−0.027 (5)

### Geometric parameters (Å, º)

**Table:**

ClA—O1A	1.389 (6)	C16—N15	1.339 (7)
ClA—O1A^i^	1.389 (6)	C16—H16	0.9500
ClA—O2A	1.402 (6)	N15—C20	1.363 (7)
ClA—O2A^i^	1.402 (6)	C35—C40	1.413 (10)
ClBA—O4BA	1.4398 (8)	C35—C34	1.427 (9)
ClBA—O1BA	1.4399 (8)	C30—H30	0.9500
ClBA—O3BA	1.4400 (8)	C27—C28	1.350 (11)
ClBA—O2BA	1.4401 (8)	C27—H27	0.9500
ClBB—O1BB	1.4399 (8)	C11—C10	1.373 (10)
ClBB—O3BB	1.4399 (8)	C11—C12	1.393 (11)
ClBB—O2BB	1.4401 (8)	C11—H11	0.9500
ClBB—O4BB	1.4402 (11)	C37—C38	1.399 (10)
ClCA—O2CA	1.4399 (8)	C37—H37	0.9500
ClCA—O4CA	1.4402 (9)	C39—C38	1.366 (13)
ClCA—O3CA	1.4405 (8)	C39—C40	1.421 (12)
ClCA—O1CA	1.4407 (8)	C39—H39	0.9500
ClCB—O4CB	1.4400 (11)	C12—C13	1.440 (10)
ClCB—O1CB	1.4400 (8)	C40—C41	1.415 (13)
ClCB—O2CB	1.4400 (8)	C21—C20	1.426 (8)
ClCB—O3CB	1.4403 (8)	C41—C42	1.347 (13)
Ru1—N22	2.053 (5)	C41—H41	0.9500
Ru1—N36	2.058 (5)	C18—C19	1.409 (9)
Ru1—N1	2.059 (5)	C18—H18	0.9500
Ru1—N15	2.063 (5)	C14—C13	1.347 (12)
Ru1—N8	2.067 (5)	C14—C5	1.426 (11)
Ru1—N29	2.068 (5)	C14—H14	0.9500
N8—C9	1.329 (8)	C2—C3	1.393 (10)
N8—C7	1.376 (8)	C2—H2	0.9500
N36—C37	1.334 (8)	C4—C3	1.365 (12)
N36—C35	1.349 (9)	C4—C5	1.387 (11)
N29—C30	1.335 (7)	C4—H4	0.9500
N29—C34	1.368 (8)	C9—C10	1.392 (8)
N22—C23	1.346 (8)	C9—H9	0.9500
N22—C21	1.383 (8)	C13—H13	0.9500
C26—C25	1.410 (10)	C6—C5	1.403 (9)
C26—C21	1.410 (9)	C19—C20	1.392 (8)
C26—C27	1.410 (11)	C19—C28	1.444 (9)
N1—C2	1.340 (9)	C38—H38	0.9500
N1—C6	1.370 (8)	C24—C23	1.409 (10)
C31—C32	1.378 (10)	C24—H24	0.9500
C31—C30	1.406 (9)	C42—C33	1.421 (11)
C31—H31	0.9500	C42—H42	0.9500
C25—C24	1.346 (11)	C33—C32	1.401 (10)
C25—H25	0.9500	C33—C34	1.409 (10)
C17—C18	1.361 (9)	C23—H23	0.9500
C17—C16	1.388 (8)	C28—H28	0.9500
C17—H17	0.9500	C10—H10	0.9500
C7—C12	1.405 (9)	C32—H32	0.9500
C7—C6	1.424 (10)	C3—H3	0.9500
			
O1A—ClA—O1A^i^	110.5 (9)	C31—C30—H30	118.9
O1A—ClA—O2A	109.5 (6)	C28—C27—C26	122.4 (6)
O1A^i^—ClA—O2A	108.3 (4)	C28—C27—H27	118.8
O1A—ClA—O2A^i^	108.3 (4)	C26—C27—H27	118.8
O1A^i^—ClA—O2A^i^	109.5 (6)	C10—C11—C12	119.2 (6)
O2A—ClA—O2A^i^	110.8 (6)	C10—C11—H11	120.4
O4BA—ClBA—O1BA	109.48 (6)	C12—C11—H11	120.4
O4BA—ClBA—O3BA	109.49 (6)	N36—C37—C38	122.9 (8)
O1BA—ClBA—O3BA	109.48 (6)	N36—C37—H37	118.5
O4BA—ClBA—O2BA	109.48 (6)	C38—C37—H37	118.5
O1BA—ClBA—O2BA	109.47 (6)	C38—C39—C40	119.4 (7)
O3BA—ClBA—O2BA	109.44 (6)	C38—C39—H39	120.3
O1BB—ClBB—O3BB	109.7 (2)	C40—C39—H39	120.3
O1BB—ClBB—O2BB	109.7 (2)	C11—C12—C7	117.5 (6)
O3BB—ClBB—O2BB	109.7 (2)	C11—C12—C13	124.4 (7)
O1BB—ClBB—O4BB	108.4 (12)	C7—C12—C13	118.0 (7)
O3BB—ClBB—O4BB	109.7 (2)	C35—C40—C41	118.4 (7)
O2BB—ClBB—O4BB	109.7 (3)	C35—C40—C39	116.7 (8)
O2CA—ClCA—O4CA	109.52 (6)	C41—C40—C39	125.0 (8)
O2CA—ClCA—O3CA	109.50 (6)	N22—C21—C26	122.5 (6)
O4CA—ClCA—O3CA	109.46 (6)	N22—C21—C20	116.3 (5)
O2CA—ClCA—O1CA	109.49 (6)	C26—C21—C20	121.1 (6)
O4CA—ClCA—O1CA	109.44 (6)	C42—C41—C40	122.2 (7)
O3CA—ClCA—O1CA	109.43 (6)	C42—C41—H41	118.9
O4CB—ClCB—O1CB	110.1 (15)	C40—C41—H41	118.9
O4CB—ClCB—O2CB	109.4 (3)	C17—C18—C19	119.2 (6)
O1CB—ClCB—O2CB	109.4 (3)	C17—C18—H18	120.4
O4CB—ClCB—O3CB	109.3 (3)	C19—C18—H18	120.4
O1CB—ClCB—O3CB	109.3 (3)	C13—C14—C5	121.6 (7)
O2CB—ClCB—O3CB	109.3 (3)	C13—C14—H14	119.2
N22—Ru1—N36	93.6 (2)	C5—C14—H14	119.2
N22—Ru1—N1	94.6 (2)	N1—C2—C3	120.6 (8)
N36—Ru1—N1	96.9 (2)	N1—C2—H2	119.7
N22—Ru1—N15	80.5 (2)	C3—C2—H2	119.7
N36—Ru1—N15	88.90 (18)	C3—C4—C5	119.2 (7)
N1—Ru1—N15	172.6 (2)	C3—C4—H4	120.4
N22—Ru1—N8	87.27 (19)	C5—C4—H4	120.4
N36—Ru1—N8	176.5 (2)	N8—C9—C10	123.2 (6)
N1—Ru1—N8	79.6 (2)	N8—C9—H9	118.4
N15—Ru1—N8	94.60 (18)	C10—C9—H9	118.4
N22—Ru1—N29	172.08 (19)	C14—C13—C12	121.3 (7)
N36—Ru1—N29	79.7 (2)	C14—C13—H13	119.4
N1—Ru1—N29	90.4 (2)	C12—C13—H13	119.4
N15—Ru1—N29	95.09 (18)	N1—C6—C5	123.4 (6)
N8—Ru1—N29	99.70 (19)	N1—C6—C7	116.3 (5)
C9—N8—C7	116.8 (5)	C5—C6—C7	120.3 (6)
C9—N8—Ru1	129.3 (4)	C20—C19—C18	117.4 (6)
C7—N8—Ru1	113.4 (4)	C20—C19—C28	118.3 (6)
C37—N36—C35	117.9 (6)	C18—C19—C28	124.3 (6)
C37—N36—Ru1	128.2 (5)	N15—C20—C19	123.1 (5)
C35—N36—Ru1	113.8 (4)	N15—C20—C21	117.1 (5)
C30—N29—C34	117.9 (5)	C19—C20—C21	119.8 (5)
C30—N29—Ru1	128.8 (4)	C39—C38—C37	119.6 (7)
C34—N29—Ru1	113.3 (4)	C39—C38—H38	120.2
C23—N22—C21	117.3 (6)	C37—C38—H38	120.2
C23—N22—Ru1	129.6 (5)	C25—C24—C23	120.2 (7)
C21—N22—Ru1	113.0 (4)	C25—C24—H24	119.9
C25—C26—C21	117.5 (7)	C23—C24—H24	119.9
C25—C26—C27	125.0 (7)	C41—C42—C33	121.3 (8)
C21—C26—C27	117.5 (6)	C41—C42—H42	119.4
C2—N1—C6	117.9 (6)	C33—C42—H42	119.4
C2—N1—Ru1	128.1 (5)	C32—C33—C34	117.3 (6)
C6—N1—Ru1	114.0 (4)	C32—C33—C42	124.5 (8)
C32—C31—C30	119.8 (6)	C34—C33—C42	118.1 (7)
C32—C31—H31	120.1	N22—C23—C24	122.4 (7)
C30—C31—H31	120.1	N22—C23—H23	118.8
C24—C25—C26	120.0 (7)	C24—C23—H23	118.8
C24—C25—H25	120.0	C27—C28—C19	120.9 (7)
C26—C25—H25	120.0	C27—C28—H28	119.6
C18—C17—C16	120.2 (6)	C19—C28—H28	119.6
C18—C17—H17	119.9	C4—C5—C6	117.1 (7)
C16—C17—H17	119.9	C4—C5—C14	124.4 (7)
N8—C7—C12	123.3 (6)	C6—C5—C14	118.5 (7)
N8—C7—C6	116.3 (6)	C11—C10—C9	119.9 (7)
C12—C7—C6	120.4 (6)	C11—C10—H10	120.0
N15—C16—C17	122.3 (5)	C9—C10—H10	120.0
N15—C16—H16	118.9	C31—C32—C33	119.4 (7)
C17—C16—H16	118.9	C31—C32—H32	120.3
C16—N15—C20	117.7 (5)	C33—C32—H32	120.3
C16—N15—Ru1	129.2 (4)	N29—C34—C33	123.2 (6)
C20—N15—Ru1	113.1 (4)	N29—C34—C35	116.1 (6)
N36—C35—C40	123.5 (6)	C33—C34—C35	120.7 (6)
N36—C35—C34	117.1 (6)	C4—C3—C2	121.5 (8)
C40—C35—C34	119.4 (7)	C4—C3—H3	119.2
N29—C30—C31	122.2 (6)	C2—C3—H3	119.2
N29—C30—H30	118.9		

Symmetry code: (i) −*x*+1, *y*, −*z*+5/2.
